# Colorectal Cancer and Central Obesity

**DOI:** 10.1001/jamanetworkopen.2024.54753

**Published:** 2025-01-16

**Authors:** Fatemeh Safizadeh, Marko Mandic, Michael Hoffmeister, Hermann Brenner

**Affiliations:** 1 Division of Clinical Epidemiology and Aging Research, German Cancer Research Center (DKFZ), Heidelberg, Germany; 2 Medical Faculty Heidelberg, Heidelberg University, Heidelberg, Germany; 3 German Cancer Consortium (DKTK), German Cancer Research Center (DKFZ), Heidelberg, Germany

## Abstract

**Importance:**

The proportion of colorectal cancer (CRC) cases attributable to excess weight, known as population attributable fraction (PAF), has been commonly based on measures of body mass index (BMI). Central obesity metrics, such as waist circumference (WC) and waist to hip ratio (WHR), are potentially better indicators of adiposity and have demonstrated stronger associations with CRC incidence.

**Objectives:**

To examine PAFs of CRC cases that are attributable to high WC and WHR and compare them to those attributable to high BMI.

**Design, Setting, and Participants:**

This population-based UK Biobank cohort study included 458 543 individuals aged 40 to 69 years at recruitment (March 2006 to July 2010) living within a reasonable distance of the 22 assessment centers across the UK. The analyses were conducted between May and July 2024.

**Exposures:**

Exposures were BMI, as a measure of general obesity, and WC and WHR, as indicators of central obesity.

**Main Outcomes and Measures:**

Hazard ratios (HRs) and corresponding 95% CIs were calculated for the associations of BMI, WC, and WHR with CRC incidence. The PAFs and 95% CIs of CRC cases attributable to high BMI, WC, and WHR were also calculated.

**Results:**

A total of 458 543 participants (median [IQR] age, 57 [50-63] years; 244 351 [53.3%] female) were included in the study. During a median follow-up of 11.8 (IQR, 10.9-12.5) years, 5944 participants were diagnosed with CRC. The HRs for the association with CRC incidence were notably smaller for BMI (HR for the highest vs lowest BMI quartile, 1.23; 95% CI, 1.14-1.33) than for WC (HR for the highest vs lowest WC quartile, 1.37; 95% CI, 1.27-1.49) and WHR (HR for the highest vs lowest WHR quartile, 1.40; 95% CI, 1.29-1.51); these associations became comparable only after accounting for possible reverse causality by excluding the initial years of follow-up. Similarly, the PAF of CRC for high BMI was 9.9% (95% CI, 5.5%-14.4%), substantially lower than the PAFs for high WC and WHR, which were 17.3% (95% CI, 12.3%-22.1%) and 17.6% (95% CI, 12.9%-22.2%), respectively. After excluding the initial 7 years of follow-up, PAF estimates became analogous across all measures of obesity and were 15.7% (95% CI, 8.9%-22.4%) for BMI, 16.9% (95% CI, 9.8%-23.8%) for WC, and 18.0% (95% CI, 11.5%-24.6%) for WHR.

**Conclusions and Relevance:**

In this cohort study of approximately half a million participants, the PAF of CRC attributable to excess weight, defined as high BMI, was considerably underestimated. The PAFs attributable to WC and WHR were consistent and much higher, underlining the importance of efforts to limit and overcome the obesity epidemic in CRC prevention.

## Key Points

**Question:**

Is the proportion of colorectal cancer cases attributable to obesity, defined by high body mass index, underestimated?

**Findings:**

In this cohort study of 458 543 individuals from a large UK Biobank cohort, the proportions of colorectal cancer cases attributable to high waist circumference and waist to hip ratio were 17.3% and 17.6%, respectively. These estimates were substantially higher compared with the corresponding estimate for high body mass index (9.9%), which increased to 15.7% when bias due to prediagnostic weight loss was addressed.

**Meaning:**

These findings suggest that central obesity metrics are likely to more comprehensively reflect the proportion of colorectal cancer cases attributable to obesity.

## Teaser

This cohort study examines the population attributable fractions of colorectal cancer that are attributable to central obesity metrics and compares them to those attributable to high body mass index in a large UK Biobank cohort.

## Introduction

The global prevalence of adult obesity has more than doubled since 1990.^1^ According to the World Health Organization, 43% of the adult population had overweight (defined as a body mass index [BMI] ≥25), and 16% had obesity (BMI ≥30) in 2022.^2^ Research from the World Cancer Research Fund (WCRF) and the International Agency for Cancer Research (IARC) demonstrates that excess weight is a well-established risk factor for various cancer types, including colorectal cancer (CRC), as evidenced by robust systematic reviews and meta-analyses.^3,4^ The proportion of CRC cases attributable to excess weight, commonly known as population attributable fraction (PAF), has been estimated to be approximately 5.8% in men and 7.0% in women worldwide in 2012^5^ and approximately 11% in Europe.^6^ Considering the increasing prevalence of overweight and obesity, the estimated PAFs are expected to be higher now and to consistently increase in the future.

However, PAFs attributable to excess BMI are likely underestimated due to the underestimation of the risk estimates for the association between high BMI and CRC incidence, which are used in PAF calculation. This underestimation could result from various factors. First, bias due to reverse causation induced by prediagnostic weight loss could contribute to this underestimation.^7^ A previous study^8^ found that substantially higher PAFs are obtained when this source of bias is addressed. Second, relying on cumulative lifetime exposure to excess BMI rather than using one-time BMI measurement results in substantially higher risk estimates of CRC incidence.^9^ Third, BMI might be a suboptimal measure of body fat compared with central obesity measures, such as waist circumference (WC) and waist to hip ratio (WHR). Body mass index does not reflect body fat distribution and may not fully capture the complex underlying biological associations between adiposity and cancer risk.^10^ Central obesity measures have shown stronger associations with CRC incidence compared with BMI in several studies.^11,12^ We aimed to calculate PAFs of CRC attributable to central obesity, using WC and WHR, as potentially more comprehensive and stronger indicators of CRC incidence, and compare them with the PAFs attributable to high BMI in the large UK Biobank cohort.

## Methods

### Data Collection

The UK Biobank prospective cohort collected data from more than a half million study participants aged 40 to 69 from March 2006 to July 2010. The assessment took place at 22 centers across England, Scotland, and Wales. Details of the UK Biobank study are described elsewhere.^13^ Briefly, the baseline assessment process included a touch screen questionnaire, face-to-face interview with a study nurse, extensive physical measurements, and collection of blood, urine, and saliva samples. All study participants provided written informed consent, and the UK Biobank study was approved by the North West Centre for Research Ethics Committee. For this cohort study, we excluded participants with a history of cancer diagnosis (according to national cancer registry records) before or at the initial assessment visit and those with missing baseline values for BMI, WC, or WHR. All analyses were performed between May and July 2024. This study followed the Strengthening the Reporting of Observational Studies in Epidemiology (STROBE) reporting guideline.

### Anthropometric Measures

Body size measures, including body weight, standing height, WC, and hip circumference, were obtained during the initial assessment center visit.^14^ Weight and standing height were measured using a body composition analyzer (BC-418 MA, Tanita Corp) and a mechanical telescopic height measuring rod (seca 202, seca GmbH & Co KG). The BMI values were calculated as weight in kilograms divided by height in meters squared. The WC and hip circumference measures were determined with a nonstretchable sprung tape measure (Wessex Medical Ltd). The WHR was calculated by dividing WC (in centimeters) by hip circumference (in centimeters).

### Cancer Incidence

Cancer data are available in the UK Biobank via linkage to the national cancer registries, and complete cancer follow-up data were available until December 31, 2020, for England, November 30, 2021, for Scotland, and December 31, 2016, for Wales according to the censoring dates provided by the UK Biobank at the time of the current analysis. CRC incidence was identified using the *International Statistical Classification of Diseases and Related Health Problems, Tenth Revision* (*ICD-10*), including cancers of the colon (C18.0-18.9), rectosigmoid junction (C19), and rectum (C20).

### Statistical Analysis

The analyses were performed using SAS software, version 9.4 (SAS Institute Inc). Baseline characteristics of the whole study population and the CRC cases were summarized using descriptive statistics. To ensure comparability, analyses for BMI, WC, and WHR were conducted using quartiles of the respective metric for the categorization (sex-specific quartiles for WC and WHR). Follow-up time was determined as the time from the first assessment visit until 1 of the following events, whichever occurred first: CRC diagnosis, loss to follow-up, or death or end of follow-up (censoring dates). Both crude and multivariable Cox proportional hazards regression models were used to estimate hazard ratios (HRs) and their corresponding 95% CIs. The multivariable-adjusted models included assessment center, age at baseline, sex, height, self-reported ethnic background (Asian or Asian British; Black or Black British; Chinese; White, including British, Irish, or any other White background; multiracial; and self-reported “other” ethnic group), Townsend deprivation index, educational qualifications, pack-years of smoking, alcohol consumption, self-reported physical activity, dietary intake, sleep duration, family history of CRC, menopausal status (women only), history of bowel cancer screening, regular use of nonsteroidal anti-inflammatory drugs, and hormone replacement therapy (HRT) (women only) (Table 1). Ethnic background was included as a variable to account for potential residual confounding because CRC risk may vary across ethnic groups due to unmeasured factors, such as genetic predisposition or culturally influenced behaviors not fully captured by the lifestyle factors already adjusted for. More information regarding the covariates is provided in eMethods 1 in Supplement 1.

**Table 1.** Baseline Characteristics of the Study Cohort^a^

Characteristic	No. (%) of participants^b^	
	Total cohort	Patients with CRC
Age at baseline, median (IQR), y	57 (50-63)	62 (56-66)
Sex		
Male	214 192 (46.7)	3405 (42.7)
Female	244 351 (53.3)	2539 (57.3)
Height, median (IQR), cm	168 (162 to 175)	170 (163 to 177)
Ethnic background^c^		
White	431 110 (94.5)	5740 (97.1)
Other	25 229 (5.5)	173 (2.9)
Townsend deprivation index, median (IQR)	−2.1 (−3.6 to 0.5)	−2.3 (−3.7 to 0.4)
Educational qualifications		
Higher academic or professional	224 480 (49.5)	2689 (45.9)
Lower academic or vocational	152 042 (33.6)	1958 (33.4)
None	76 593 (16.9)	1215 (20.7)
BMI, median (IQR)	26.7 (24.2 to 29.9)	27.3 (24.7 to 30.4)
WC, median (IQR), cm	90.0 (81.0 to 99.0)	94.0 (84 to 102)
WHR, median (IQR)	0.87 (0.80 to 0.94)	0.91 (0.83 to 0.96)
Pack-years of smoking, median (IQR), y	0.0 (0.0 to 11.0)	0.0 (0.0 to 19.9)
Alcohol consumption		
Never	93 176 (20.4)	1557 (26.3)
Special occasion only	106 422 (23.3)	1370 (23.1)
1-3 times a month	118 367 (25.9)	1386 (23.4)
Once or twice a week	50 981 (11.1)	545 (9.2)
3-4 times a week	52 117 (11.4)	637 (10.7)
Daily or almost daily	36 458 (8.0)	434 (7.3)
Physical activity (IPAQ groups)		
Low	64 909 (18.4)	843 (18.6)
Moderate	143 279 (40.6)	1908 (42.1)
High	144 780 (41.0)	1785 (39.4)
Favorable dietary intake		
Fruits ≥3	227 995 (49.9)	2860 (48.3)
Vegetables ≥3	372 060 (81.8)	4856 (82.5)
Processed meats ≤1	313 866 (68.7)	3861 (65.1)
Unprocessed red meats ≤1.5	299 003 (66.0)	3627 (61.7)
Fish ≥2	236 201 (51.7)	3194 (53.9)
Whole grains ≥3	50 334 (11.1)	698 (11.9)
Refined grains ≤1.5	325 570 (72.1)	3992 (68.2)
Sleep duration, median (IQR), h/d	7 (7 to 8)	7 (7 to 8)
History of bowel cancer screening		
No	312 956 (69.5)	3748 (64.0)
Yes	137 093 (30.5)	2107 (36.0)
Family history of CRC		
No	399 743 (89.0)	4922 (84.4)
Yes	49 344 (11.0)	907 (15.6)
Regular use of NSAIDs or aspirin		
No	317 913 (69.3)	4075 (68.6)
Yes	140 614 (30.7)	1869 (31.4)
Menopausal status (women only)		
Premenopausal	60 495 (26.6)	295 (12.1)
Postmenopausal	167 170 (73.4)	2139 (87.9)
HRT use (women only)		
No	151 518 (62.3)	1339 (53.0)
Yes	91 570 (37.7)	1187 (47.0)

Abbreviations: BMI, body mass index (calculated as weight in kilograms divided by height in meters squared); CRC, colorectal cancer; HRT, hormone replacement therapy; IPAQ, International Physical Activity Questionnaire; NSAIDs, nonsteroidal anti-inflammatory drugs.

^a^ Numbers of variable missing values in total cohort are as follows: age (0), sex (0), height (0), ethnic background (2204), Townsend deprivation index (564), educational qualifications (5428), pack-years of smoking (70 673), alcohol consumption (1022), physical activity (105 575), dietary intake (614), sleep duration (3443), bowel cancer screening (8494), family history of CRC (9456), NSAID use (16), menopausal status (16 686), and HRT use (1263). Percentages may not total 100% due to rounding.

^b^ Unless otherwise indicated.

^c^ White ethnic background included British, Irish, or any other White background, and other ethnic backgrounds included Asian or Asian British, Black or Black British, Chinese, multiracial, and self-reported “other” ethnic group.

The analyses were conducted including complete follow-up (0-14 years) and excluding the first 2, 4, or 7 years of follow-up (ie, including follow-up times of 2-14, 4-14, or 7-14 years) to account for the potential underestimation of HRs caused by prediagnostic weight loss.^8^ The analysis using the crude model was performed only for the complete follow-up. Additionally, to evaluate the robustness of our findings, we calculated the E-values for our main analysis, which quantify the minimum strength of association on the risk ratio scale, that an unmeasured confounder would need to have with both the obesity measure and the CRC risk to fully explain away the observed associations.^15^

The proportional hazards assumption was investigated by Schoenfeld residuals graphs, and no violation was detected. Missing values were imputed using the multiple imputation procedure PROC MI, and the obtained 5 imputed datasets were then combined using the PROC MIANALYZE. All variables had less than 2% missing values except physical activity, which had approximately 20% missing values. Age and sex had no missing values.

### Subgroup Analysis

Subgroup-specific analyses were performed by age group (<60 vs ≥60 years), sex (men vs women), and smoking status (never smoker vs ever smoker). Potential modification by the stratification variables was evaluated by including an interaction term of each anthropometric measure (continuous) and the stratification variables in the model. We also performed site-specific analyses for colon and rectal cancers separately, and potential heterogeneity of associations with both outcomes was assessed using the heterogeneity test. In all analyses, 2-sided *P* < .05 was considered statistically significant.

### Sensitivity Analysis

To specifically evaluate the effect of dietary intake and physical activity on the results, we conducted the main analysis for complete follow-up without adjusting for these 2 variables and compared them with the results from the fully adjusted model. Additionally, to better understand the association between central obesity and CRC risk, we performed a sensitivity analysis evaluating the association between WHR adjusted for BMI (continuous), alongside the covariates included in the fully adjusted model, and CRC for follow-up periods of 0 to 14, 2 to 14, 4 to 14, and 7 to 14 years.

### PAF Calculation

The PAFs (95% CIs) of CRC cases attributable to adiposity defined as increased BMI, WC, and WHR were calculated for 0- to 14-year and 7- to 14-year follow-up periods, using the graphPAF R package PAF_calc_discrete() function with 1000 bootstrap replications in R, version 4.4 (R Foundation).^16,17^ The graphPAF R package calculates PAFs incorporating the aforementioned multivariable Cox proportional hazards regression models and using the direct method, which estimates PAFs by summing estimated probabilities of disease in the absence of exposure on the individual level (eMethods 2 in Supplement 1). As previously mentioned, quartiles of BMI, WC, and WHR (sex-specific quartiles for WC and WHR) were used for PAF calculations. The reference value was specified as the lowest quartile, and any excess risk for quartiles 2, 3, and 4 was considered accordingly.

## Results

**Figure 1.**
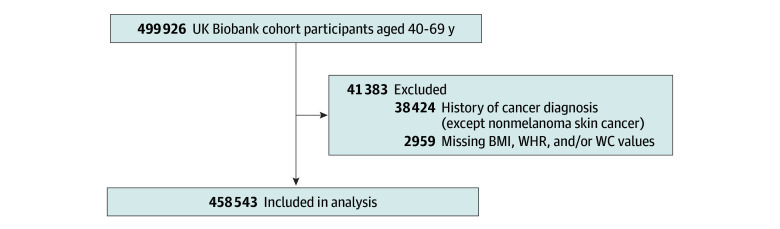
Study Population Flow Diagram BMI indicates body mass index; WC, waist circumference; WHR, waist to hip ratio.

Table 1 displays the baseline characteristics of the total cohort and CRC cases. Our analysis included 458 543 UK Biobank participants (Figure 1), with a median age of 57 (IQR, 50-63) years, of whom 244 351 (53.3%) were female, 214 192 (46.7%) were male, 431 110 (94.5%) were White, and 25 229 (5.5%) were other races (Asian or Asian British, Black or Black British, Chinese, multiracial, and other ethnic groups). In the total population, the median BMI was 26.7 (IQR, 24.2-29.9), WC was 90.0 (IQR, 81.0-99.0) cm, and WHR was 0.87 (IQR, 0.80-0.94). Of the female participants, 167 170 (73.4%) were postmenopausal and 91 570 (37.7%) were using HRT. During a median (IQR) follow-up of 11.8 (10.9-12.5) years, 5944 participants were diagnosed with CRC. Participants diagnosed with CRC were older, had lower levels of education, and had higher BMI, WC, and WHR at baseline. They also more often had a family history of CRC, and female participants with incident CRC were more often postmenopausal and HRT users than those without CRC diagnosis during follow-up.

Table 2 presents the HRs for the association of BMI, WC, and WHR with CRC incidence risk, including complete follow-up and after excluding the first 2, 4, and 7 years of follow-up. For BMI, the HRs for CRC incidence risk increased as more initial follow-up years were excluded. The HRs for the highest quartile vs the lowest quartile were 1.23 (95% CI, 1.14-1.33) when complete follow-up was included in the analysis and 1.27 (95% CI, 1.16-1.38), 1.29 (95% CI, 1.18-1.41), and 1.37 (95% CI, 1.22-1.53) after excluding the initial 2, 4, and 7 years of follow-up, respectively. Unlike for BMI, the HRs for WC and WHR remained highly consistent regardless of follow-up years included in the analysis and similar to those for BMI after excluding the first 7 years of follow-up. The HRs for the association between the highest vs lowest quartile of WC and CRC risk were 1.37 (95% CI, 1.27-1.49) when complete follow-up years were considered and 1.38 (95% CI, 1.27-1.50), 1.36 (95% CI, 1.23-1.49), and 1.40 (95% CI, 1.24-1.57) after excluding the first 2, 4, and 7 years of follow-up. For WHR, the HRs for the association between the highest vs lowest quartile and CRC risk were 1.40 (95% CI, 1.29-1.51) for the complete follow-up years and 1.43 (95% CI, 1.31-1.55), 1.40 (95% CI, 1.28-1.54), and 1.43 (95% CI, 1.28-1.61) after excluding the first 2, 4, and 7 years of follow-up.

**Table 2.** HRs for Incident Colorectal Cancer Risk Associated With General and Central Obesity Measures by Follow-Up Periods

Follow-up, y	No. of cases	HR (95% CI) by quartile^a^			
		First	Second	Third	Fourth
**BMI** ^b^					
0-14	5944	1 [Reference]	1.05 (0.97-1.13)	1.14 (1.06-1.23)	1.23 (1.14-1.33)
2-14	5155	1 [Reference]	1.04 (0.96-1.14)	1.14 (1.05-1.24)	1.27 (1.16-1.38)
4-14	4232	1 [Reference]	1.07 (0.97-1.17)	1.15 (1.05-1.26)	1.29 (1.18-1.41)
7-14	2773	1 [Reference]	1.12 (1.00-1.26)	1.23 (1.10-1.38)	1.37 (1.22-1.53)
**WC**^c^					
0-14	5944	1 [Reference]	1.13 (1.05-1.23)	1.26 (1.16-1.36)	1.37 (1.27-1.49)
2-14	5155	1 [Reference]	1.09 (1.00-1.19)	1.24 (1.14-1.35)	1.38 (1.27-1.50)
4-14	4232	1 [Reference]	1.08 (0.98-1.19)	1.23 (1.12-1.36)	1.36 (1.23-1.49)
7-14	2773	1 [Reference]	1.08 (0.95-1.21)	1.27 (1.13-1.43)	1.40 (1.24-1.57)
**WHR**^d^					
0-14	5944	1 [Reference]	1.15 (1.07-1.25)	1.24 (1.15-1.34)	1.40 (1.29-1.51)
2-14	5155	1 [Reference]	1.15 (1.05-1.25)	1.24 (1.14-1.35)	1.43 (1.31-1.55)
4-14	4232	1 [Reference]	1.16 (1.05-1.27)	1.21 (1.11-1.33)	1.40 (1.28-1.54)
7-14	2773	1 [Reference]	1.17 (1.05-1.32)	1.22 (1.09-1.37)	1.43 (1.28-1.61)

Abbreviations: BMI, body mass index (calculated as weight in kilograms divided by height in meters squared); HR, hazard ratio; WC, waist circumference; WHR, waist to hip ratio.

^a^ Adjusted for assessment center, age, sex, height, ethnicity, socioeconomic deprivation, educational level, pack-years of smoking, alcohol consumption, physical activity, dietary intake, sleep duration, history of bowel cancer screening, family history of colorectal cancer, menopausal status (women only), hormone replacement therapy use (women only), and regular use of nonsteroidal anti-inflammatory drugs.

^b^ For BMI, quartiles are as follows: first quartile, less than 24.2; second quartile, 24.2 to less than 26.7; third quartile, 26.7 to less than 29.9; and fourth quartile, 29.9 or greater.

^c^ For WC, quartiles are as follows: first quartile, less than 89 cm for men and less than 75 cm for women; second quartile, 89 to less than 96 cm for men and 75 to less than 83 cm for women; third quartile, 96 to less than 103 cm for men and 83 to less than 92 cm for women; and fourth quartile, 103 cm or greater for men and 92 cm or greater for women.

^d^ For WHR, quartiles are as follows: first, less than 0.89 for men and less than 0.77 for women; second, 0.89 to less than 0.93 for men and 0.77 to less than 0.81 for women; third, 0.93 to less than 0.98 for men and 0.81 to less than 0.86 for women; and fourth, 0.98 or greater for men and 0.86 or greater for women.

The results from models excluding physical activity and dietary intake were similar and, alongside the results from unadjusted models, are shown in eTable 1 in Supplement 1. Furthermore, similar to WHR and WC, WHR adjusted for BMI showed a stronger and more consistent association with CRC risk compared with BMI (eTable 2 in Supplement 1). Additionally, the E-values calculated for the association between anthropometric measures and CRC risk indicated that our findings are relatively robust to unmeasured confounding (eTable 3 in Supplement 1).

The results of the subgroup analysis by age group, sex, and smoking status and site-specific analysis for colon and rectal cancers are given in Table 3 and the eFigure in Supplement 1 for the entire follow-up period and after excluding the initial 7 years. The observed associations followed a similar pattern to the main analysis. The HRs for the association between BMI and CRC incidence increased when prediagnostic weight loss was accounted for and sufficient initial follow-up years were excluded. Contrarily, even before excluding the initial follow-up years, the HRs for WC and WHR were comparable to those observed for BMI after such exclusions. These robust associations for WC and WHR persisted after excluding the early follow-up years. The HRs were higher among men compared with women and for colon cancer compared with rectal cancer for all adiposity measures. Tests for interaction showed statistically significant results for sex across all anthropometric measures (*P* < .001 for interaction) and for age category specifically for WHR (*P* = .003 for interaction). The heterogeneity test for colon vs rectal cancer was not statistically significant for BMI (*P* = .55 for heterogeneity), WC (*P* = .31 for heterogeneity), or WHR (*P* = .23 for heterogeneity).

**Table 3.** Subgroup and Site-Specific HRs for Incident Colorectal Cancer Risk Associated With General and Central Obesity Measures

Follow-up, y	No. of cases	Quartiles, HR (95% CI)^a^			
		First	Second	Third	Fourth
**BMI (quartiles)** ^b^					
Age <60 y					
0-14	2152	1 [Reference]	1.04 (0.92-1.18)	1.12 (0.99-1.27)	1.25 (1.10-1.41)
7-14	1072	1 [Reference]	1.07 (0.89-1.28)	1.11 (0.93-1.33)	1.38 (1.16-1.64)
Age ≥60 y					
0-14	3792	1 [Reference]	1.07 (0.97-1.18)	1.18 (1.07-1.30)	1.23 (1.11-1.36)
7-14	1701	1 [Reference]	1.18 (1.02-1.38)	1.33 (1.15-1.54)	1.37 (1.17-1.59)
Men					
0-14	3405	1 [Reference]	1.03 (0.92-1.15)	1.25 (1.12-1.39)	1.39 (1.24-1.56)
7-14	1562	1 [Reference]	1.09 (0.92-1.29)	1.33 (1.13-1.56)	1.52 (1.28-1.79)
Women					
0-14	2539	1 [Reference]	1.12 (1.00-1.24)	1.04 (0.93-1.16)	1.06 (0.95-1.19)
7-14	1211	1 [Reference]	1.21 (1.04-1.42)	1.13 (0.96-1.33)	1.21 (1.03-1.42)
Never smoker					
0-14	2739	1 [Reference]	0.99 (0.89-1.10)	1.08 (0.97-1.20)	1.22 (1.09-1.37)
7-14	1287	1 [Reference]	1.11 (0.94-1.30)	1.17 (1.00-1.38)	1.33 (1.13-1.57)
Ever smoker					
0-14	3181	1 [Reference]	1.10 (0.98-1.23)	1.19 (1.07-1.33)	1.24 (1.11-1.38)
7-14	1478	1 [Reference]	1.13 (0.96-1.33)	1.26 (1.08-1.48)	1.38 (1.17-1.62)
Colon cancer					
0-14	3988	1 [Reference]	1.08 (0.98-1.19)	1.19 (1.08-1.30)	1.30 (1.18-1.43)
7-14	1873	1 [Reference]	1.17 (1.01-1.34)	1.29 (1.12-1.48)	1.40 (1.22-1.61)
Rectal cancer					
0-14	1975	1 [Reference]	0.99 (0.87-1.13)	1.07 (0.94-1.22)	1.11 (0.97-1.27)
7-14	899	1 [Reference]	1.05 (0.86-1.29)	1.15 (0.94-1.40)	1.31 (1.07-1.60)
**WC (sex-specific quartiles)** ^c^					
Age <60 y					
0-14	2152	1 [Reference]	1.23 (1.08-1.40)	1.40 (1.24-1.60)	1.50 (1.31-1.70)
7-14	1072	1 [Reference]	1.11 (0.93-1.33)	1.32 (1.10-1.59)	1.64 (1.37-1.96)
Age ≥60 y					
0-14	3792	1 [Reference]	1.11 (1.00-1.23)	1.24 (1.12-1.38)	1.38 (1.24-1.53)
7-14	1701	1 [Reference]	1.08 (0.92-1.27)	1.30 (1.11-1.51)	1.34 (1.15-1.56)
Men					
0-14	3405	1 [Reference]	1.19 (1.06-1.33)	1.40 (1.25-1.56)	1.55 (1.39-1.73)
7-14	1562	1 [Reference]	1.10 (0.94-1.30)	1.36 (1.16-1.59)	1.53 (1.31-1.79)
Women					
0-14	2539	1 [Reference]	1.07 (0.95-1.21)	1.10 (0.98-1.24)	1.16 (1.03-1.31)
7-14	1211	1 [Reference]	1.05 (0.88-1.25)	1.17 (0.98-1.39)	1.22 (1.03-1.46)
Never smoker					
0-14	2739	1 [Reference]	1.15 (1.02-1.28)	1.24 (1.11-1.40)	1.37 (1.22-1.54)
7-14	1287	1 [Reference]	1.05 (0.89-1.23)	1.25 (1.06-1.47)	1.36 (1.15-1.60)
Ever smoker					
0-14	3181	1 [Reference]	1.11 (0.99-1.25)	1.25 (1.11-1.40)	1.35 (1.21-1.51)
7-14	1478	1 [Reference]	1.10 (0.92-1.31)	1.27 (1.08-1.51)	1.41 (1.20-1.67)
Colon cancer					
0-14	3988	1 [Reference]	1.14 (1.03-1.26)	1.28 (1.16-1.41)	1.45 (1.31-1.60)
7-14	1873	1 [Reference]	1.10 (0.95-1.28)	1.30 (1.12-1.50)	1.48 (1.28-1.71)
Rectal cancer					
0-14	1975	1 [Reference]	1.14 (0.99-1.31)	1.24 (1.08-1.42)	1.25 (1.08-1.43)
7-14	899	1 [Reference]	1.04 (0.85-1.28)	1.26 (1.03-1.54)	1.25 (1.02-1.53)
**WHR (sex-specific quartiles)** ^d^					
Age <60 y					
0-14	2152	1 [Reference]	1.20 (1.06-1.35)	1.34 (1.19-1.52)	1.48 (1.30-1.68)
7-14	1072	1 [Reference]	1.16 (0.98-1.38)	1.27 (1.07-1.51)	1.59 (1.34-1.90)
Age ≥60 y					
0-14	3792	1 [Reference]	1.17 (1.05-1.30)	1.26 (1.14-1.40)	1.45 (1.31-1.61)
7-14	1701	1 [Reference]	1.23 (1.05-1.44)	1.26 (1.08-1.47)	1.45 (1.25-1.69)
Men					
0-14	3405	1 [Reference]	1.24 (1.11-1.38)	1.40 (1.26-1.56)	1.57 (1.42-1.75)
7-14	1562	1 [Reference]	1.29 (1.10-1.51)	1.39 (1.19-1.63)	1.59 (1.36-1.86)
Women					
0-14	2539	1 [Reference]	1.06 (0.95-1.20)	1.07 (0.95-1.20)	1.20 (1.06-1.34)
7-14	1211	1 [Reference]	1.05 (0.88-1.24)	1.02 (0.86-1.21)	1.24 (1.05-1.47)
Never smoker					
0-14	2739	1 [Reference]	1.17 (1.05-1.31)	1.28 (1.15-1.43)	1.49 (1.34-1.67)
7-14	1287	1 [Reference]	1.22 (1.04-1.43)	1.24 (1.05-1.45)	1.51 (1.28-1.78)
Ever smoker					
0-14	3181	1 [Reference]	1.12 (1.00-1.26)	1.17 (1.05-1.31)	1.29 (1.16-1.44)
7-14	1478	1 [Reference]	1.11 (0.94-1.32)	1.17 (0.99-1.38)	1.33 (1.13-1.57)
Colon cancer					
0-14	3988	1 [Reference]	1.14 (1.04-1.26)	1.23 (1.12-1.35)	1.42 (1.30-1.57)
7-14	1873	1 [Reference]	1.20 (1.04-1.39)	1.22 (1.06-1.41)	1.50 (1.30-1.72)
Rectal cancer					
0-14	1975	1 [Reference]	1.20 (1.04-1.37)	1.28 (1.12-1.47)	1.35 (1.18-1.55)
7-14	899	1 [Reference]	1.14 (0.93-1.39)	1.23 (1.01-1.50)	1.32 (1.08-1.62)

Abbreviations: BMI, body mass index (calculated as weight in kilograms divided by height in meters squared); HR, hazard ratio; WC, waist circumference; WHR, waist to hip ratio.

^a^ Adjusted for assessment center, age, sex, height, ethnicity, socioeconomic deprivation, educational level, pack-years of smoking, alcohol consumption, physical activity, dietary intake, sleep duration, history of bowel cancer screening, family history of colorectal cancer, menopausal status (women only), hormone replacement therapy use (women only), and regular use of nonsteroidal anti-inflammatory drugs.

^b^ For BMI, quartiles are as follows: first quartile, less than 24.2; second quartile, 24.2 to less than 26.7; third quartile, 26.7 to less than 29.9; and fourth quartile, 29.9 or greater.

^c^ For WC, quartiles are as follows: first quartile, less than 89 cm for men and less than 75 cm for women; second quartile, 89 to less than 96 cm for men and 75 to less than 83 cm for women; third quartile, 96 to less than 103 cm for men and 83 to less than 92 cm for women; and fourth quartile, 103 cm or greater for men and 92 cm or greater for women.

^d^ For WHR, quartiles are as follows: first, less than 0.89 for men and less than 0.77 for women; second, 0.89 to less than 0.93 for men and 0.77 to less than 0.81 for women; third, 0.93 to less than 0.98 for men and 0.81 to less than 0.86 for women; and fourth, 0.98 or greater for men and 0.86 or greater for women.

**Figure 2.**
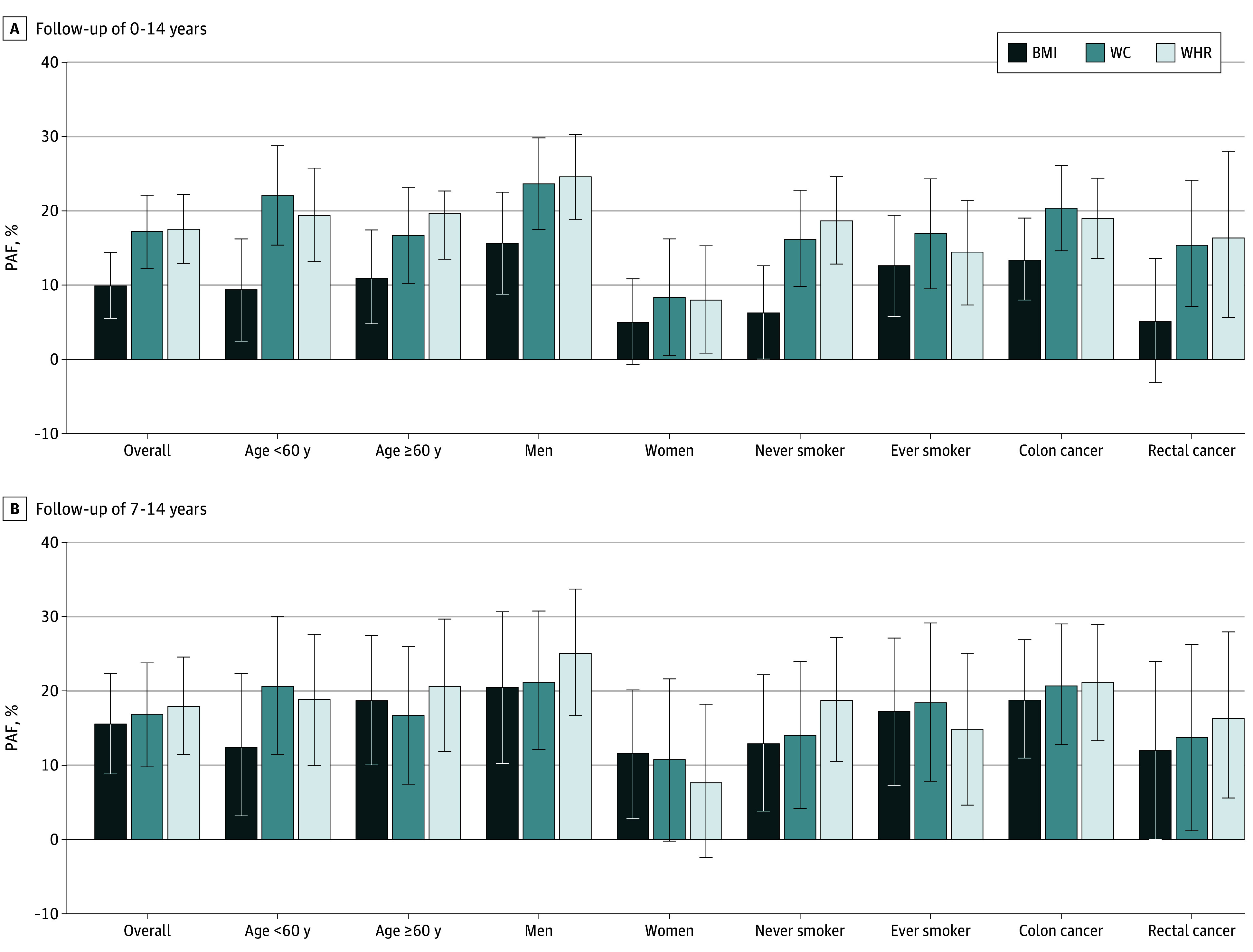
Population Attributable Fractions (PAFs) of Colorectal Cancer Cases Attributable to High Body Mass Index (BMI), Waist Circumference (WC), and Waist to Hip Ratio (WHR) Error bars indicate 95% CIs.

Figure 2 illustrates PAFs (95% CIs) of CRC cases attributable to adiposity defined as high BMI, WC, and WHR with complete follow-up and after excluding 7 years of follow-up. As shown, PAFs were small (approximately 10%) when high BMI was used as the measure of adiposity and complete follow-up years were considered, whereas they were considerably higher when WC and WHR were used. The PAFs of CRC attributable to high BMI increased when the initial follow-up years were excluded and became similar to those attributable to high WC and WHR. This trend was consistent for all subgroups of participants and for site-specific PAFs for colon and rectal cancers. For men and women combined, overall PAFs of CRC cases attributable to BMI, WC, and WHR were 9.9% (95% CI, 5.5%-14.4%), 17.3% (95% CI, 12.3%-22.1%), and 17.6% (95% CI, 12.9%-22.2%), respectively, with complete follow-up and 15.7% (95% CI, 8.9%-22.4%), 16.9% (95% CI, 9.8%-23.8%), and 18.0% (95% CI, 11.5%-24.6%), respectively, after excluding the first 7 years of follow-up. The PAFs were particularly strong for men, reaching levels of 20.6% (95% CI, 10.3%-30.7%), 21.3% (95% CI, 12.2%-30.8%), and 25.1% (95% CI, 16.8%-33.8%) for BMI, WC, and WHR, respectively, after excluding the first 7 years of follow-up.

## Discussion

In our study, we demonstrated that PAFs of CRC due to excess weight may be substantially higher than previously estimated PAFs, which mostly relied on BMI measurements. According to estimates within the UK Biobank population, 15% to 20% of overall CRC cases may be attributable to excess weight, with much higher proportions among men (approximately 20%-25%) than among women (approximately 10%). These PAF estimates based on WHR and WC were consistent across the entire period of follow-up, whereas much lower estimates would be obtained for BMI if the initial years of follow-up were not excluded.

A plausible explanation for these patterns might be underestimation of the BMI and CRC associations during the initial years of follow-up due to reverse causality resulting from cancer-related cachexia. Cancer cachexia, a prevalent syndrome among patients with cancer, is characterized by muscle loss with or without fat mass loss.^18^ In patients with gastrointestinal cancers, approximately 75% of this weight loss occurs before diagnosis.^19^ Given that BMI does not distinguish between fat mass and muscle mass, it is more vulnerable to reflecting muscle mass loss, which is a key feature of cancer cachexia. By contrast, WC and WHR are much less affected by loss of muscle mass. Consequently, the associations between high WC and WHR and, hence, the estimated proportion of CRC cases attributable to central obesity might be less prone to bias due to prediagnostic weight loss.

The PAF of CRC cases attributable to high BMI, estimated at 9.9% (95% CI, 5.5%-14.4%) in our study before accounting for prediagnostic weight loss aligns closely with findings from another UK Biobank study, which reported a PAF of CRC incidence attributable to a BMI of 25 or greater at 9.4% (95% CI, 7.3%-11.7%).^20^ However, for reasons outlined above and as demonstrated in our analysis, the true contribution of excess weight to the burden of CRC is likely substantially higher when accounting for prediagnostic weight loss in the analysis.

In contrast to more extensive research available for cardiovascular diseases, the evidence regarding PAF of cancer cases, particularly CRC cases attributable to central obesity, is limited in the literature. In a study from 2015, the burden of cancer attributable to excess body weight (BMI ≥25) and central adiposity considered as high WC (≥94 cm in men and ≥80 cm in women) and high WHR (≥0.90 in men and ≥0.85 in women) was evaluated for Canada.^21^ Relative risks were extracted from WCRF/AICR reports and published meta-analyses (which are likely to have been affected by cancer-related weight loss^7^), whereas prevalence data for obesity measures were sourced from national health surveys. In the previous work,^21^ PAFs of CRC cases attributable to excess abdominal adiposity and high WHR were 8.0% and 10.6%, respectively. The PAFs of CRC attributable to excess weight were reported separately for colon and rectal cancer at 6.9% and 3.7%, respectively. Our finding of higher PAFs for measures of central obesity than for BMI is consistent with these results, but absolute values are higher for all measures of excess weight. Apart from differences in the prevalence of these measures, a plausible explanation for the higher values derived in our study is that excess risk of CRC was considered for all participants in quartiles 2 to 4 of the BMI, WC, and WHR compared with the bottom quartile. As can be seen in our results in Table 2, there was a clear dose-response relationship between all 3 metrics and CRC risk, with increased risks already seen in the second compared with the first quartile (ie, in a range that includes participants who would typically be classified as having [upper] normal rather than excess weight). For example, the second BMI quartile ranged from 24.2 to 26.7 (ie, it included participants with BMIs between 24.2 and 25.0, who would generally be classified as having normal weight). Our results suggest, however, that PAFs exclusively based on body weight cutoffs that have not been specifically defined for CRC risk might somewhat underestimate the fraction of CRC cases that may be attributable to higher weight. Apart from the overall higher levels of PAFs disclosed in our study, our findings are consistent with previous research regarding stronger associations and higher PAFs for colon than for rectal cancer and, in particular, for men than for women.^4,5,6,7,8,9,10,11,12,13,14,15,16,17,18,19,20,21,22^

As for CRC, studies estimating PAFs for excess weight based on measures other than BMI are sparse for other cancers. Nevertheless, similar results have been found in studies on pancreatic cancer,^23,24^ showing higher PAFs attributable to central obesity, defined by increased WC and WHR, compared with those attributable to overweight and obesity, defined as high BMI. Further research should more comprehensively consider measures of central adiposity when evaluating PAFs of cancer due to excess weight.

### Strengths and Limitations

This study’s strengths include the use of the large and comprehensive UK Biobank dataset, which ensured high statistical power in our analyses and allowed for the adjustment of a wide range of covariates in our models. Moreover, all anthropometric measures used in our study were obtained through standardized methods within the UK Biobank study, eliminating any potential biases from self-reporting. Nonetheless, it is important to thoroughly discuss and consider several limitations that are present in this study. First, we tried to minimize the underestimation of PAFs attributable to obesity. However, we were unable to address all the potential sources of bias, for instance, including cumulative lifetime exposure to obesity measures, which might have led to an underestimation in our PAF estimates.^9,10,11,12,13,14,15,16,17,18,19,20,21,22,23,24,25^ We also relied on 1-time measurements of BMI and other obesity indicators because data regarding changes in anthropometric measures during follow-up were not available. Furthermore, we were unable to adjust for repeated-measures and time-varying variables because this information is available for only a small subset of UK Biobank participants. Evidence of healthy volunteer bias in the UK Biobank cohort suggests that participants are more health conscious and less socioeconomically deprived than the general population within the same age group.^26^ Consequently, the prevalence of excess weight and central obesity might be lower than in the general population, suggesting that the derived PAFs may underestimate PAFs in the general population. Moreover, although we adjusted for the Townsend deprivation index, this index may not fully reflect the impact of income on health outcomes, and data on lifetime alcohol consumption were unavailable. Furthermore, we cannot rule out residual confounding. Finally, given that 94% of the participants were White, our findings might not be generalizable to other populations.

## Conclusions

In this cohort study of approximately 500 000 participants, we demonstrated that calculating the proportion of CRC cases attributable to central obesity, as opposed to the routine approach of using BMI, provided more accurate estimates of the PAFs of obesity-related CRC cases. On the basis of our results, higher proportions of CRC cases were attributable to excess weight than commonly assumed, and central obesity had greater relevance regarding obesity-related CRC incidence. Central obesity measures showed a stronger and more robust association with CRC incidence compared with BMI. We recommend incorporating central obesity measures, such as WC and WHR, alongside BMI, which does not account for fat mass distribution, in estimating the burden of CRC due to excess weight and the potential for prevention. Our PAF findings underline the importance of efforts to limit and overcome the obesity epidemic in CRC prevention.

## Notes

Supplement 1. **eMethods 1.** Covariate Assessment **eMethods 2.** PAF Calculation **eTable 1.** Unadjusted Hazard Ratios (HRs), Fully Adjusted HRs, and Fully Adjusted HRs Excluding Dietary Intake and Physical Activity, Along With Their 95% Confidence Intervals (CIs), for Incident Colorectal Cancer Risk Associated With General and Central Obesity Measures Over Complete Follow-Up Time **eTable 2.** Hazard Ratios (HRs) and Their 95% Confidence Intervals (CIs) for Incident Colorectal Cancer Risk Associated With Waist-To-Hip Ratio (WHR) Adjusted for Body Mass Index (BMI) Including Various Follow-Up Time Windows **eTable 3.** E-Values for the Associations Between BMI, WC, and WHR With Colorectal Cancer Risk **eFigure**. Subgroup and Site-Specific Hazard Ratios (HRs) and Their 95% Confidence Intervals (CIs) for Incident Colorectal Cancer Risk Associated With BMI, WC, and WHR Including Complete Follow-Up Years (0-14) and After Excluding the Initial 7 Years of Follow-Up (>7-14)
eReferences

Supplement 2.
Data Sharing Statement
