# Supplementary material for: Colorectal Cancer and Central Obesity
Source: JAMA Netw Open. 2025 Jan 16;8(1):e2454753. doi: 10.1001/jamanetworkopen.2024.54753 (PMC11739990; doi:10.1001/jamanetworkopen.2024.54753)
Supplement: Supplement 1. — eMethods 1. Covariate Assessment eMethods 2. PAF Calculation eTable 1. Unadjusted Hazard Ratios (HRs), Fully Adjusted HRs, and Fully Adjusted HRs Excluding Dietary Intake and Physical Activity, Along With Their 95% Confidence Intervals (CIs), for Incident Colorectal Cancer Risk Associated With General and Central Obesity Measures Over Complete Follow-Up Time eTable 2. Hazard Ratios (HRs) and Their 95% Confidence Intervals (CIs) for Incident Colorectal Cancer Risk Associated With Waist-To-Hip Ratio (WHR) Adjusted for Body Mass Index (BMI) Including Various Follow-Up Time Windows eTable 3. E-Values for the Associations Between BMI, WC, and WHR With Colorectal Cancer Risk eFigure. Subgroup and Site-Specific Hazard Ratios (HRs) and Their 95% Confidence Intervals (CIs) for Incident Colorectal Cancer Risk Associated With BMI, WC, and WHR Including Complete Follow-Up Years (0-14) and After Excluding the Initial 7 Years of Follow-Up (>7-14) eReferences [file jamanetwopen-e2454753-s001.pdf]

Supplementary Online Content

Safizadeh F, Mandic M, Hoffmeister M, Brenner H. Colorectal cancer and central obesity. *JAMA Netw Open*. 2024;8(1):e2454753. doi:10.1001/jamanetworkopen.2024.54753

eMethods 1. Covariate Assessment

eMethods 2. PAF Calculation

eTable 1. Unadjusted Hazard Ratios (HR), Fully Adjusted HRs, and Fully Adjusted HRs Excluding Dietary Intake and Physical Activity, Along With Their 95% Confidence Intervals (CI), for Incident Colorectal Cancer Risk Associated With General and Central Obesity Measures Over Complete Follow-Up Time

eTable 2. Hazard Ratios (HR) and Their 95% Confidence Intervals (CI) for Incident Colorectal Cancer Risk Associated With Waist-to-Hip Ratio (WHR) Adjusted for Body Mass Index (BMI) Including Various Follow-Up Time Windows

eTable 3. E-Values for the Associations Between BMI, WC, and WHR With Colorectal Cancer Risk

eFigure. Subgroup and Site-Specific Hazard Ratios (HR) and Their 95% Confidence Intervals (CI) for Incident Colorectal Cancer Risk Associated With BMI, WC, and WHR Including Complete Follow-Up Years (0-14) and After Excluding the Initial 7 Years of Follow-Up (>7-14)

eReferences

This supplementary material has been provided by the authors to give readers additional information about their work.

© 2025 Safizadeh F et al. *JAMA Network Open.*

eMethods 1. Covariate Assessment

Townsend deprivation index: The Townsend Deprivation Index was assigned to participants based on their postal code, with scores corresponding to the census output area where each participant resided. The index was calculated immediately before participants joined the UK Biobank, and was derived from four census-based variables: unemployment rate, car ownership, home ownership, and household crowding.

Physical activity: Physical activity was assessed in the UK Biobank at baseline using adapted questions from the validated short-form International Physical Activity Questionnaire (IPAQ),[1] which evaluates the frequency, intensity, and duration of different types of activities. These include walking, moderate activities (such as carrying light loads, cycling at a regular pace, or playing doubles tennis), and vigorous activities (like heavy lifting, digging, aerobics, or fast cycling). The questionnaire also captures sedentary time, including sitting at a desk, visiting friends, reading, or watching television, at work, home, or during leisure time.

Dietary intake: A diet score was calculated based on self-reported intake frequencies for fruit (fresh and dried), vegetable (raw, cooked), processed meat, unprocessed red meat (beef, lamb, pork), fish (oily, non-oily), whole grains (bran cereal, oat cereal, whole meal or wholegrain bread), and refined grains (biscuit cereal, muesli, cornflakes, Frosties, white and brown bread). This diet score is based on the dietary priorities for cardiovascular health and has been previously applied to assess the relationship between dietary patterns and colorectal cancer risk.[2] A healthy diet was defined by the following criteria: fruit intake ≥3 servings/day, vegetable intake ≥3 servings/day, processed meat intake ≤1 serving/week, unprocessed red meat intake ≤1.5 servings/week, fish intake ≥2 servings/week, whole grain intake ≥3 servings/day, and refined grain intake ≤1.5 servings/day. One point was assigned for each food component where the healthy diet criteria were met, with zero points given otherwise. The total score ranged from 0 to 7 and was categorized as low (0–1 points), intermediate (2–5 points), and high (6–7 points).

© 2025 Safizadeh F et al. *JAMA Network Open.*

eMethods 2. PAF calculation

PAFs (95% CI) of CRC cases attributable to adiposity defined as increased BMI, WC, and WHR were calculated for two follow-up time windows: 0-14 and 7-14 years, using the graphPAF R package[3-4] PAF_calc_discrete() function with 1000 bootstrap replications in R version 4.4. The graphPAF R package calculates PAFs incorporating the aforementioned multivariable Cox proportional hazards models, and using the direct method, which estimates PAFs by summing estimated probabilities of disease in the absence of exposure on the individual level. Specifically, the formula used for PAF calculation is:

$$PAF = \frac{\sum_{i=1}^{n} P_i(RR_i - 1)}{1 + \sum_{i=1}^{n} P_i(RR_i - 1)}$$

where P_i represents the proportion of the population in exposure category *i*, quartiles of BMI, WC, and WHR (sex-specific quartiles for WC and WHR), and RR_i is the estimated hazard ratio (HR) for that category relative to the reference category. The sum of PAFs across the exposure categories gives the total PAF. The reference value was specified as the lowest quartile, and any excess risk for quartiles 2, 3, and 4 was considered accordingly.

Assumptions:

1- The potential outcome, which represents the condition in which the risk factor has been eliminated, must be defined and measured with precision and clarity.

2- The assessment of a set of covariates is conducted such that, for a given observed value of the covariates, the probability of the outcome occurring under the condition that the risk factor is absent is equal to the probability of the outcome occurring in the absence of the risk factor, given the covariates. This equivalence holds true when the risk factor behaves as if it were assigned randomly within the strata defined by the covariates. This collection of covariates is often referred to as a sufficient adjustment set.

3. The model, conditioned on the risk factor and covariates, is correctly specified.

© 2025 Safizadeh F et al. *JAMA Network Open.*

eTable 1. Unadjusted hazard ratios (HR), fully adjusted HRs, and fully adjusted HRs excluding dietary intake and physical activity, along with their 95% confidence intervals (CI), for incident colorectal cancer risk associated with general and central obesity measures over complete follow-up time.

**Anthropometric measures**	**N cases**	**Unadjusted HR (95% CI)**	**Adjusted HR[a] (95% CI)**	**Adjusted HR[a] (95% CI) excluding dietary intake and physical activity**
**Body mass index (kg/m^2)**				
<24.2	1,196	Ref.	Ref.	Ref.
24.2-<26.7	1,436	1.21 (1.12-1.30)	1.05 (0.97-1.13)	1.05 (0.97-1.13)
26.7-<29.9	1,645	1.39 (1.29-1.49)	1.14 (1.06-1.23)	1.15 (1.07-1.24)
≥29.9	1,667	1.42 (1.32-1.53)	1.23 (1.14-1.33)	1.25 (1.15-1.35)
**Waist circumference (cm)**				
Men: <89 Women: <75	983	Ref.	Ref.	Ref.
Men: 89-<96, Women: 75-<83	1,437	1.25 (1.15-1.36)	1.13 (1.05-1.23)	1.14 (1.05-1.24)
Men: 96-<103, Women: 83-<92	1,592	1.49 (1.37-1.61)	1.26 (1.16-1.36)	1.27 (1.17-1.37)
Men: ≥103, Women: ≥92	1,932	1.68 (1.56-1.82)	1.37 (1.27-1.49)	1.39 (1.29-1.51)
**Waist-to-hip ratio**				
Men: <0.89, Women: <0.77	1,081	Ref.	Ref.	Ref.
Men: 0.89-<0.93, Women: 0.77-<0.81	1,376	1.29 (1.19-1.40)	1.15 (1.07-1.25)	1.16 (1.07-1.26)
Men: 0.93-<0.98, Women: 0.81-<0.86	1,612	1.50 (1.39-1.62)	1.24 (1.15-1.34)	1.25 (1.16-1.35)
Men: ≥0.98, Women: ≥0.86	1,875	1.81 (1.68-1.95)	1.40 (1.29-1.51)	1.42 (1.31-1.53)

[a]Adjusted for assessment center, age, sex, height, ethnicity, socio-economic deprivation, education, pack-years of smoking, alcohol consumption, physical activity, dietary intake, sleep duration, history of bowel cancer screening, family history of CRC, menopausal status (women only), HRT use (women only), and regular use of NSAIDs.

Abbreviations: *BMI* Body Mass Index; *CI* Confidence Interval; *HR* Hazard Ratio; *WC* Waist circumference; *WHR* Waist-to-hip ratio.

© 2025 Safizadeh F et al. *JAMA Network Open.*

eTable 2. Hazard ratios (HR) and their 95% confidence intervals (CI) for incident colorectal cancer risk associated with waist-to-hip ratio (WHR) adjusted for body mass index (BMI) including various follow-up time windows.

**Included years of FU**	**N cases**	**HR[a] (95% CI)**			
**WHR (sex-specific quartiles)**		**Q1** Men:<0.89, Women:<0.77	**Q2** Men:0.89-<0.93, Women:0.77-<0.81	**Q3** Men:0.93-<0.98, Women:0.81-<0.86	**Q4** Men:≥0.98, Women:≥0.86
0-14	5,944	Ref.	1.13 (1.04-1.23)	1.20 (1.10-1.30)	1.32 (1.21-1.44)
2-14	5,155	Ref.	1.13 (1.03-1.23)	1.19 (1.09-1.30)	1.34 (1.22-1.47)
4-14	4,232	Ref.	1.13 (1.03-1.24)	1.16 (1.05-1.28)	1.30 (1.17-1.44)
7-14	2,773	Ref.	1.14 (1.01-1.28)	1.15 (1.02-1.29)	1.30 (1.15-1.47)

[a]Adjusted for assessment center, age, sex, height, **BMI**, ethnicity, socio-economic deprivation, education, pack-years of smoking, alcohol consumption, physical activity, dietary intake, sleep duration, history of bowel cancer screening, family history of CRC, menopausal status (women only), HRT use (women only), and regular use of NSAIDs.

Abbreviations: *BMI* Body Mass Index; *CI* Confidence Interval; *HR* Hazard Ratio; *WHR* Waist-to-hip ratio.

© 2025 Safizadeh F et al. *JAMA Network Open.*

eTable 3. E-values for the associations between BMI, WC, and WHR with colorectal cancer risk.

Included years of FU	E-value			
**BMI (quartiles/kg/m^2)**	**Q1**<br><24.2	**Q2**<br>24.2-<26.7	**Q3**<br>26.7-<29.9	**Q4**<br>≥29.9
0-14	------------	1.28	1.54	1.76
2-14	------------	1.24	1.54	1.86
4-14	------------	1.34	1.57	1.90
7-14	------------	1.49	1.76	2.08
**WC (sex-specific quartiles/cm)**	**Q1**<br>Men:<89<br>Women:<75	**Q2**<br>Men:89-<96<br>Women:75-<83	**Q3**<br>Men:96-<103,<br>Women:83-<92	**Q4**<br>Men:≥103,<br>Women:≥92
0-14	------------	1.51	1.83	2.08
2-14	------------	1.40	1.79	2.10
4-14	------------	1.37	1.76	2.06
7-14	------------	1.37	1.86	2.15
**WHR (sex-specific quartiles)**	**Q1**<br>Men:<0.89,<br>Women:<0.77	**Q2**<br>Men:0.89-<0.93,<br>Women:0.77-<0.81	**Q3**<br>Men:0.93-<0.98,<br>Women:0.81-<0.86	**Q4**<br>Men:≥0.98,<br>Women:≥0.86
0-14	------------	1.57	1.79	2.15
2-14	------------	1.57	1.79	2.21
4-14	------------	1.59	1.71	2.15
7-14	------------	1.62	1.74	2.21

Abbreviations: *BMI* Body Mass Index; *CI* Confidence Interval; *HR* Hazard Ratio; *WC* Waist circumference; *WHR* Waist-to-hip ratio.

© 2025 Safizadeh F et al. *JAMA Network Open.*

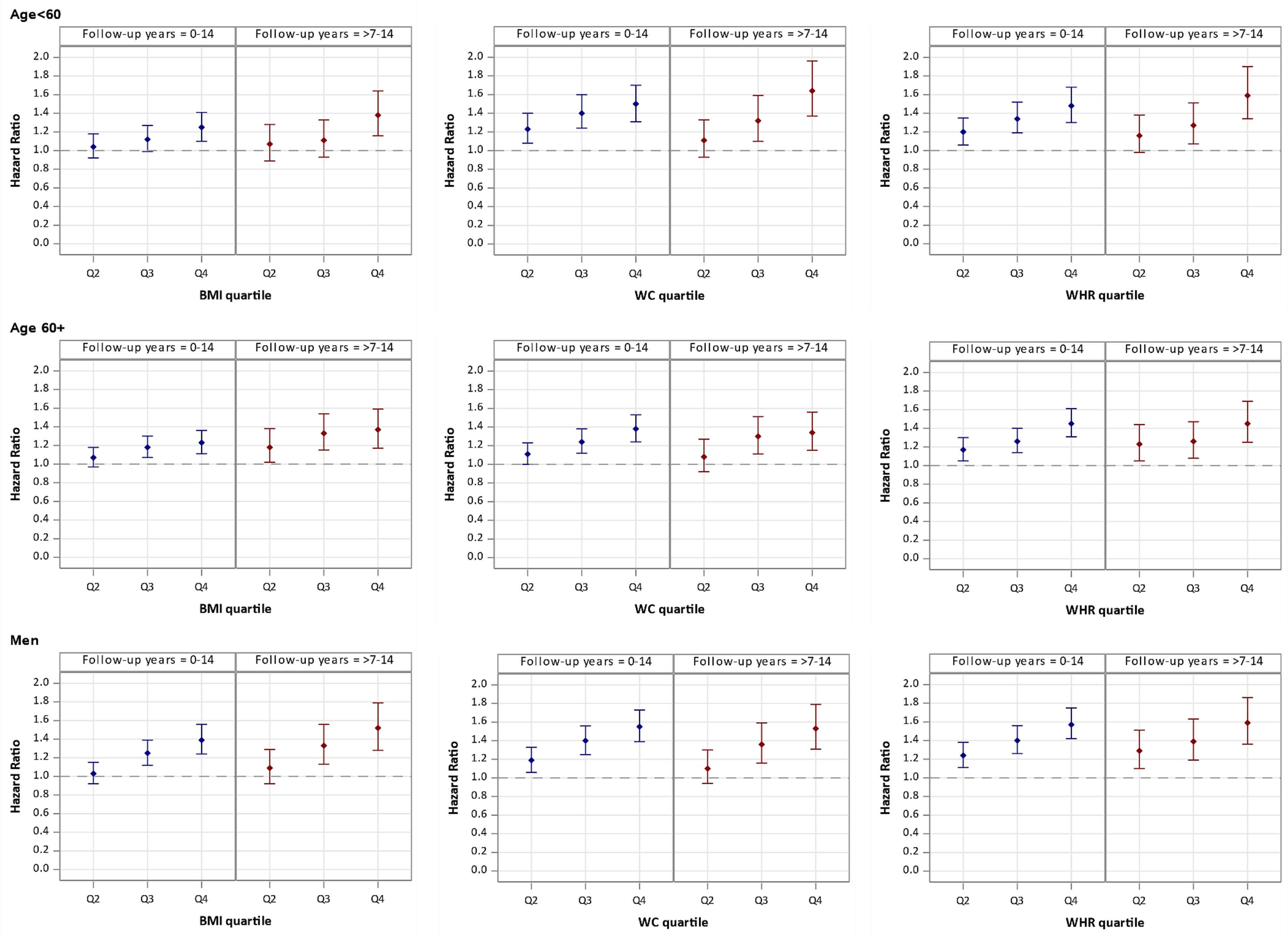

© 2025 Safizadeh F et al. *JAMA Network Open.*

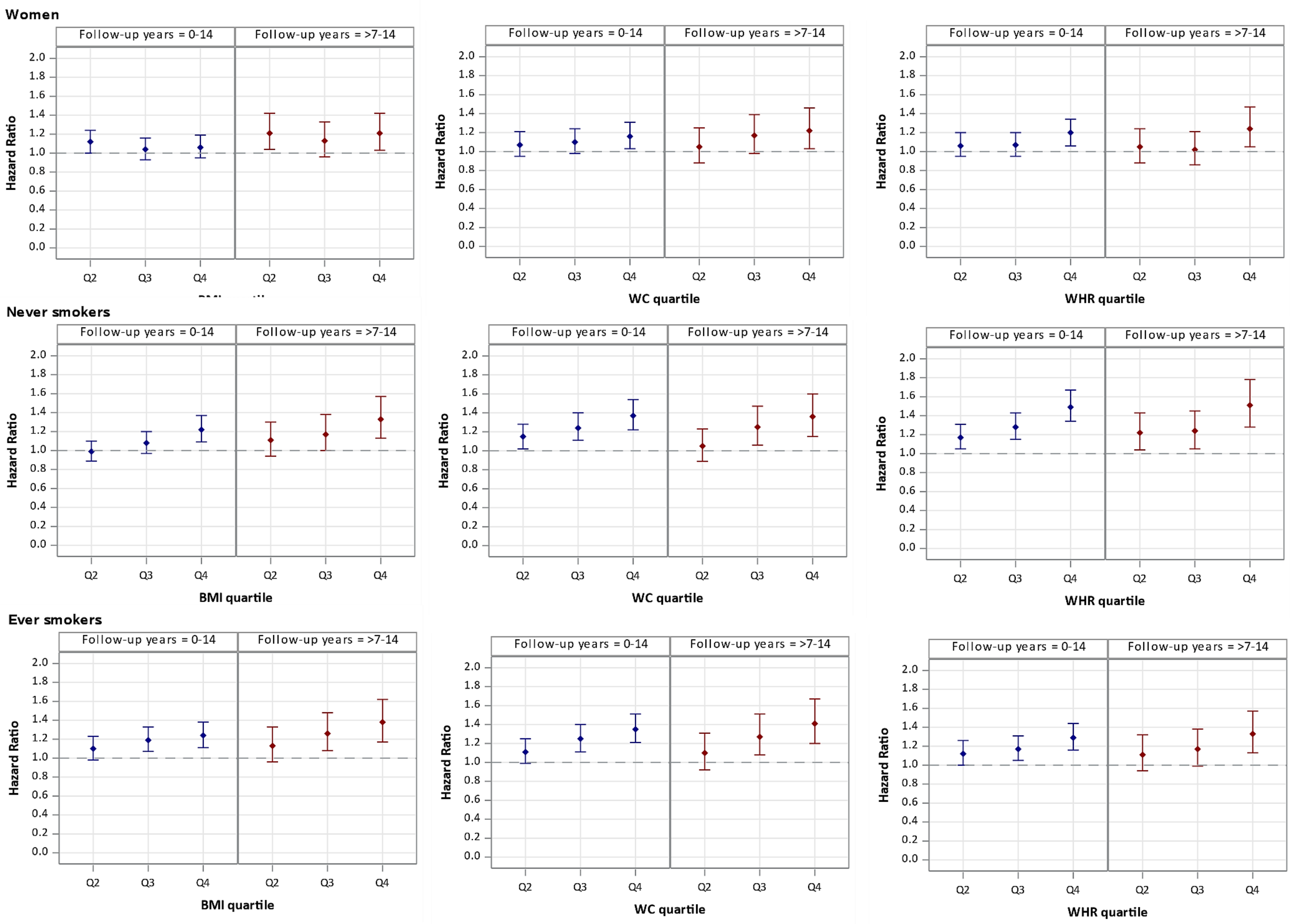

© 2025 Safizadeh F et al. *JAMA Network Open.*

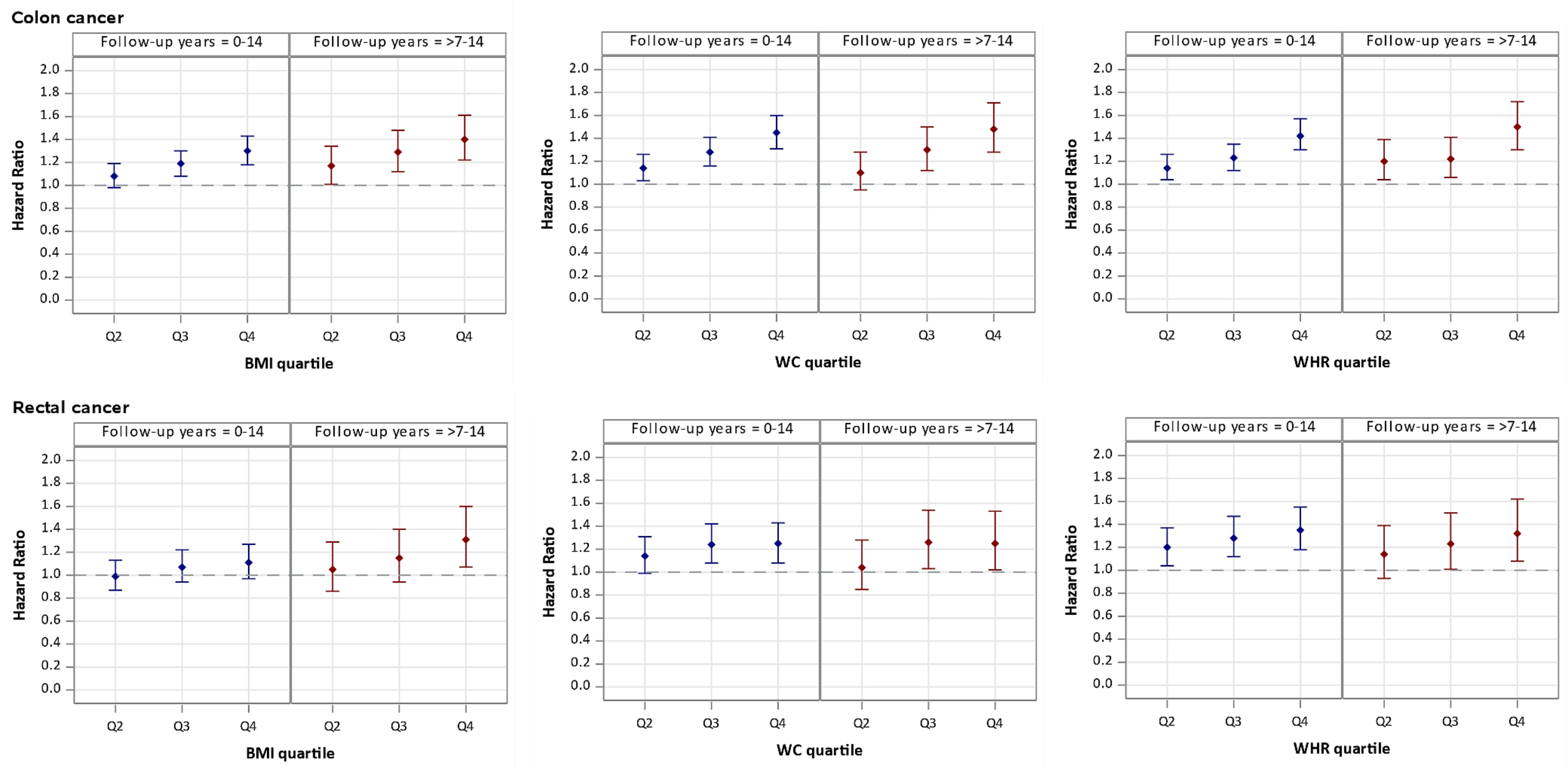

eFigure. Subgroup and site-specific hazard ratios (HR) and their 95% confidence intervals (CI) for incident colorectal cancer risk associated with BMI, WC, and WHR including complete follow-up years (0-14) and after excluding the initial 7 years of follow-up (>7-14).

The models were adjusted for assessment center, age, sex, height, ethnicity, socio-economic deprivation, education, pack-years of smoking, alcohol consumption, physical activity, dietary intake, sleep duration, history of bowel cancer screening, family history of CRC, menopausal status (women only), HRT use (women only), and regular use of NSAIDs.

BMI (kg/m^2) cut-offs were:.24.2, 26.7, and 29.9. WC (cm) cut-offs were: Men: 89, 96, and 103, Women: 75, 83, and 92. WHR cut-offs were: Men: 0.89, 0.93, and 0.98, Women: 0.77, 0.81, and 0.86.

© 2025 Safizadeh F et al. *JAMA Network Open.*

eReferences

1. Craig CL, Marshall AL, Sjöström M, et al. International physical activity questionnaire: 12-country reliability and validity. Med Sci Sports Exerc. 2003;35(8):1381-95. doi:10.1249/01.Mss.0000078924.61453.Fb

2. Huang J, Ye E, Li X, et al. Association of healthy diet score and adiposity with risk of colorectal cancer: findings from the UK Biobank prospective cohort study. Eur J Nutr. 2024;63(6):2055-69. doi:10.1007/s00394-024-03418-7

3. Ferguson J. Package 'graphPAF'. Available at: https://cran.r-project.org/web/packages/graphPAF/graphPAF.pdf accessed on 10/06/2024.

4. Ferguson J, O'Connell M. graphPAF: An R package to estimate and display population attributable fractions. Available at: https://cran.r-project.org/web/packages/graphPAF/vignettes/graphPAF_vignette.pdf accessed on 10/06/2024.

© 2025 Safizadeh F et al. *JAMA Network Open.*
