# Supplementary material for: Colorectal Cancer and Central Obesity
Source: JAMA Netw Open. 2025 Jan 16;8(1):e2454753. doi: 10.1001/jamanetworkopen.2024.54753 (PMC11739990; doi:10.1001/jamanetworkopen.2024.54753)
Supplement: Supplement 2. — Data Sharing Statement [file jamanetwopen-e2454753-s002.pdf]

Data Sharing Statement

Safizadeh,. Colorectal Cancer and Central Obesity. *JAMA Netw Open*. Published January 16, 2025. doi:10.1001/jamanetworkopen.2024.54753

Data
Data available: No
